# Burn wounds infected with Pseudomonas aeruginosa triggers weight loss in rats

**DOI:** 10.1186/1471-2482-5-19

**Published:** 2005-09-17

**Authors:** Lars Steinstraesser, Olaf Burkhard, Ming H Fan, Frank Jacobsen, Marcus Lehnhardt, Grace Su, Adrien Daigeler, Hans U Steinau, Daniel Remick, Stewart C Wang

**Affiliations:** 1 Dept. of Surgery, University of Michigan, 1150 W. Medical Center Drive, Ann Arbor, MI 48109-0666, USA; 2 Medicine, University of Michigan, 1150 W. Medical Center Drive, Ann Arbor, MI 48109-0666, USA; 3 Pathology, University of Michigan, 1150 W. Medical Center Drive, Ann Arbor, MI 48109-0666, USA; 4 Dept. Plastic Surgery Burn Center, Ruhr-University Bochum, Buerkle-de la-Camp Platz 1, 44789 Bochum, Germany

## Abstract

**Background:**

Despite dramatic improvements in the management of burns, infection still remains a serious risk for the burn patient. The aim of this study was to shed light on the impact of acute burn injury with or without infection on cytokine profiles.

**Methods:**

Sprague-Dawley rats (n = 21) were randomized into three groups: 1) burn only 2) burn and infection or 3) sham burn. Weight was monitored and blood was collected for cytokine ELISA, LPS quantification, and peripheral blood analysis. Animals were sacrificed either after 6 or 12 days.

**Results:**

Infected animals showed substantial weight loss until day 6 post-burn as compared to burn alone. Endotoxin and TNF-α levels were elevated early in the infected burn group within 48 hours post-burn. In contrast, significant up-regulation of the anti-inflammatory cytokine IL-10 occurred later in the clinical course and was associated with the recovery from weight loss.

**Conclusion:**

Our results suggest that in the presence of infection, you get a SIRS response possibly due to transient endotoxemia that is only seen in the infection group. In contrast, both burn and infection get a late IL-10 (CARS) response, which is then associated with a return to normal weight in the infection group.

## Background

While current procedures for burn injury management have improved patient prognosis, increased morbidity and mortality remains a major challenge for the clinician. Thus, identification of the mechanisms responsible for post-burn immune dysfunction and increased susceptibility to wound infection, subsequent sepsis and multiple organ failure under such conditions, is crucial for the development of improved treatment modalities. Thermal injury induces an immuncompromised state that predisposes patients to sepsis and multiple organ failure [1-6]. Skin, as the first line of defense against invading microbes, is equipped with an array of immune mediators capable of recruiting inflammatory cells to enable neutralization and clearance of bacteria and fungi [7,8]. Hence, immune failure in a burn patient who has lost the skin barrier is vulnerable to infection. These are major complications associated with burn trauma and recent evidence suggests that activation of a pro-inflammatory cascade plays an important role in their development [9].

For a long time the integument had been considered as an organ of passive protection but over the past few years several discoveries have shown that the skin is not only the target of diseases connected with immunological mechanisms but moreover, an important immunocompetent organ in itself [10]. The difficulty depends on the causal connectivity of findings since a clear distinction has not always been made between the early shock phase and the later continuance of organ failure. Various physiologic and immunologic alterations are observed in acute inflammations associated with infection, trauma and thermal injury. Recently, increasing attention has been directed to the role of cytokines in the mechanisms of such alterations. Tumor necrosis factor-α (TNF-α), interleukin-1β (IL-1β), interleukin-6 (IL-6), which are pro-inflammatory cytokines, and interleukin-10 (IL-10) are considered potential mediators of inflammation produced by immunoregulatory cells as well as by a variety of other cell types [11]. Cytokines play a major role in the regulation of immune response, hematopoesis and inflammation [12].

The aim of this paper is to shed light on the role of wound infection with *Pseudomonas aeruginosa *on the systemic inflammatory response and weight loss in burn injury.

## Methods

### Bacteria

A multi drug resistant strain of *Pseudomonas aeruginosa *isolated from human burn wound was used for this study. Over night culture was diluted into fresh TSB and incubated for 2.5 h at 37°C. The subculture was centrifuged (10 min, 4°C, 880 *g*). The bacterial pellet was washed once and resuspended in a cold sodium phosphate buffer (J.T.Baker, Deventer, Holland, pH 7.4). CFU were calculated by OD_600 _nm measurement (UV-VIS-Spektrometer, Perkin Elmer 555).

### Animals

Adult male Sprague-Dawley rats (250–300 g) were obtained from the Unit for Laboratory Animal Medicine (University of Michigan, Ann Arbor, MI) and maintained under standard laboratory conditions. In addition to a resting period at the institutional vivarium, animals were acclimated to the laboratory environment for at least 48 hours before treatment. After treatment, the rats were placed in individual cages in a temperature controlled room with food and water provided ad libitum and a 12 hour light and a 12 hour dark diurnal cycle. All experiments were performed in accordance with National Institute of Health guidelines and approval was obtained from the University of Michigan Animal Care and Use Committee.

### Experimental design

Sprague-Dawley rats (n = 21) were randomized into three groups: 1) burn only; 2) burn and infection (10^8 ^CFU of multi-drug resistant *Pseudomonas aeruginosa*) or 3) sham burn. Animals in the first two groups received a 30% (TBSA) partial thickness burn. The animals were anesthetized prior to each intervention with intraperitoneal Ketamine hydrochloride (100 mg/kg; Fort Dodge Laboratories, Fort Dodge, Iowa) and Xylazine (13 mg/kg; Bayer Corporation, Shawnee Mission, Kansas) injection. The skin of the whole torso was clipped and treated with depilatory cream (Sally Hansen^®^Div. Del Laboratories, Inc., Farmingdale. NY). Twenty-four hours later, rats were placed in a mold, which exposed an area of 30% total body surface area (TBSA). Rats, except the sham burn group 3, sustained a superficial partial scald injury at the defined area on both flanks and back at 60°C for twenty seconds. This extend of injury in this animal model is time dependent and has been previously described [13]. Additionally, the burn wound of the infected group was covered with a 2 cm × 2 cm gauze containing 10^8 ^CFU (colony forming units) of log-phase *Pseudomonas aeruginosa*. Injury was covered with Tegaderm™ HP (3 M Health Care, St. Paul, MN) and Flex-Wrap™ Self-Adherent Wrap (The Kendall Company, Mansfield, MA). After treatment, rats were resuscitated subcutaneously with 5 ml saline. For the remainder of the study, all animals received buprenorphine (0.3 mg/kg) intra peritoneal twice daily for pain control post-burn. In previous experiments we have not observed any abdominal injury with this scald burn model which could affect food intake or absorption [13-17]. Weight changes were monitored daily. Blood was collected after 12, 24, 48 and 72 h, then after 6 and 12 days. This was used for cytokine ELISA, LPS quantification, and peripheral blood analysis. After either 6 or 12 days the animals were sacrificed and treated areas of the infected and non infected wound tissues were harvested aseptically, weighed, homogenized, serially diluted and plated in triplicate on trypticase soy agar with 5% sheep blood and *Pseudomonas *isolation agar (both from Becton Dickinson). Bacterial plates were then incubated for 18 hours and the number of colony forming units were counted in blinded fashion. Results are expressed as CFU per gram infected skin tissue.

### Peripheral blood analysis

At appropriate time points, 20 μl of EDTA anti-coagulated blood was collected from the rat tail vein. A Hemavet Mascot Multispecies Hematology System Counter 1500 R (CDC Technologies Inc., Oxford, CT) was used to measure the blood differential, WBC, RBC, hemoglobin and hematocrit. Furthermore, 200 μl of plasma was collected and stored at -20°C for cytokine and endotoxin screening.

### Endotoxin levels

For the purpose of determining the amount of endotoxin present in the plasma, a QCL-1000 Limulus Amebocyte Lysate Assay (Bio Whittaker Inc., Walkersville, MD) was performed. This assay uses a modified Limulus Amebocyte Lysate and a coloring substrate to detect gram-negative bacterial endotoxin chromogenically. Following the manufacturer's instruction, 50 μl of plasma was diluted 1:10 in order to quantify the endotoxin levels. Concentrations were photospectrometrically analyzed at 410 nm and calculated corresponding to the standard curve.

### Cytokines

With the purpose of quantifying changes in cytokine levels, ELISA's were performed for IL-1β, IL-6, Il-10 and TNF-α using Cytoscreen™ Immunoassay Kits (BioSource International, Inc., Camarillo, CA). Following the manufacturer's instruction, 50 μl of plasma was used in a 1:1 dilution. ELISA plates were read on a plate reader (Bio-Tek Instruments, Windoski, VT) at dual wavelengths of 465 nm and 590 nm. Sample concentrations were determined by comparison to a standard curve.

### Statistical analysis

Data with normal distribution was analyzed using ANOVA and Student's *t*-test (StatView^®^, Abacus Concepts/SAS^® ^Institute, Cary, N.C.). Results were considered to be significant at p < 0.05 and expressed as the mean ± SEM.

## Results

### Wound infection

Macroscopic evaluation of burned skin immediately after the scald injury revealed notable paleness. A few minutes later a reddish pink coloration appeared, giving way to progressive pallor over the next 30–60 minutes. Edema reached a peak after approximately 2 hours and then slowly regressed, disappearing completely during the ensuing 24 hours. At the day of sacrifice, the infected wounds showed clear signs of macroscopic wound infection with pus and redness in the inoculated and adjacent tissue. Tissue counts in the infected group showed 10^6^-10^7 ^CFU of *P. aeruginosa *per gram of tissue compared to 10^1^-10^2 ^CFU in the non infected groups. Throughout the study period no mortalities in any of the groups was observed.

### Weight changes

**Figure 1:**
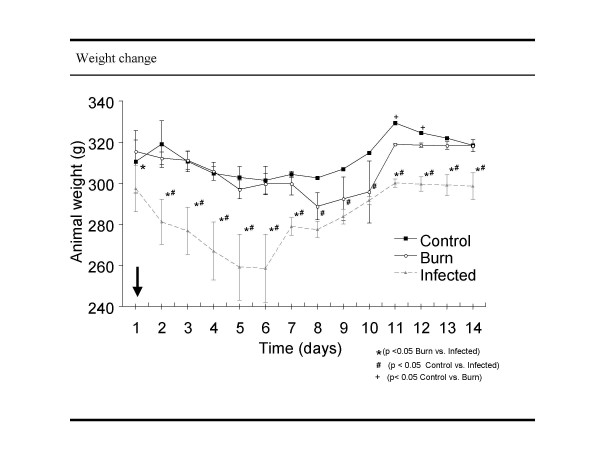
**Animal body weight. **Body weight of each animal was measured every day during the whole experiment.

Weight loss is a prominent metabolic response to thermal injury and infection. As shown in figure 1, weight loss was significant higher for 6 days postoperatively in the infected burn group compared to the other treatment groups. In the infected group, weight decreases constantly until day 6 (mean difference day 1–6: 38.820 g). In this period the weight loss shows significance (p < 0.05) compared to the control group at every time point. Although the infected burn group recovers from the first weight loss and regained weight after 7 days postoperatively, weight remains significantly lower than in the control group. No significant difference in weight loss was observed comparing the sham burn groups with the burn only group. Differences in food intake or direct metabolic effects comparing the burn and the infected burn group have not been observed.

### Peripheral blood analysis

Lymphocytes were significantly lower after 12 hours post treatment for the burn (5000/ml ± 900; p = 0.04) and the infected group (3000/ml ± 500; p = 0.02) compared to no treatment control (9000/ml ± 800). No significant differences were observed for neutrophils, monocytes, red blood cell count, platelets, hemoglobin and hematocrit (data not shown).

### Endotoxin levels

**Figure 2:**
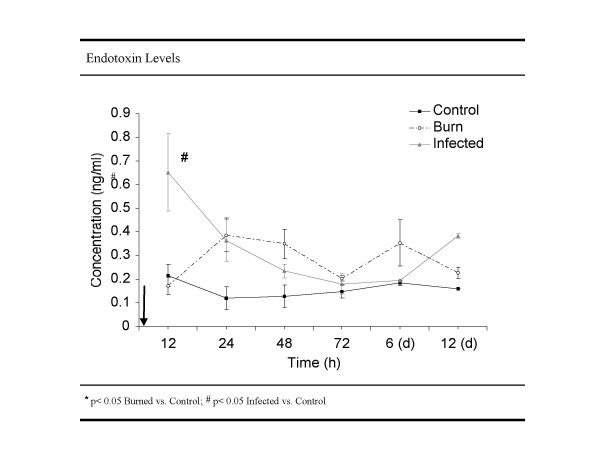
**Endotoxin level. **Blood serum endotoxin levels were measured 12, 24, 48, 72 h, 6 and 12 days post burn injury in infected and control group (each n = 6). Endotoxin amount was calculated in ng/ml serum. The values at 12 h, 6 and 12 days were further displayed as bar graph to demonstrate significant (p < 0,05) differences.

In the infected animals, endotoxin levels were significantly higher 12 hours post treatment for the infected burn group (625 pg/ml ± 200) compared to the burn (172 pg ± 30; p = 0.02) and control (216 pg/ml ± 48; p = 0.02) group with no difference comparing burn only with the control group. 24 hours post treatment no difference between the groups was noticeable (Figure 2).

### Cytokines

#### IL-1

**Figure 3:**
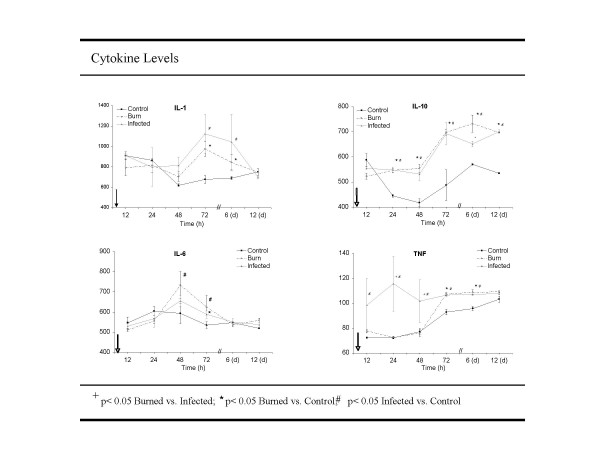
**Cytokine expression. **Blood was obtained from each rat in the separated treatment groups after 24, 48 and 72 h, 6 and 12 days post injury. Serum was separated and analyzed for IL-1β, IL-6, IL-10 and TNF-α using ELISA detection system. A time course is shown including the whole follow up of the study. Amount cytokine expression is displayed in pg/ml serum.

Until 48 hours after thermal injury, IL-1 levels were detected in comparable concentrations (Figure 3). After 72 hours, IL-1 concentrations in the infected group (1.125 ng/ml ± 0.19) and in the burn group (0.997 ng/ml ± 0.07) peaked and remained significantly (p = 0.03) higher compared to the no treatment control group until they returned to baseline after 12 days.

#### IL-6

In the burn group and infected burn group, IL-6 concentrations showed a peak 48 h postburn followed by a descend (p < 0.02) reaching baseline levels on day 6 compared to no treatment control (Figure 3). No significant difference was observed comparing the burn vs. infected burn group.

#### IL-10

*Between 24 and 72 hours postburn IL-10 levels increased significantly in the burn and infected burn group with a steady state after the 6^th ^day postburn *(Figure 3). No significant difference was seen comparing the burn vs. infected burn group.

#### TNF-α

Levels for TNF-α were significantly higher 48 h postburn in the infected burn group compared *to the burn and no treatment control group*. The initial endotoxemia is followed by 24 and 48 hr TNF rise which is then followed by gradual weight loss in the infection vs. burn alone group. *After 48 hours *TNF-α concentrations of the infected and burn groups were comparable and significantly higher compared to the control group until day 12 (p < 0,01).

## Discussion

Extensive burn trauma induces an acute inflammatory response that is associated with a variety of systemic alterations including increased vascular permeability, myocardial dysfunction, hypermetabolism and altered hepatic synthetic activity [18-21].

This study demonstrates significantly higher TNF-α and endotoxin levels associated with substantial higher weight loss in infected burn wounds compared to non-infected burn wounds. In the utilized animal model, weight loss was observed for 6 days post burn in the infected group, whereas no differences in weight loss were observed in the non-infected burn group. However, no pair feeding was carried out within this study in order to explain differences in weight gain or loss. In a clinical setting it is impossible to investigate the reason for hypermetabolism in burn patients [22].

Previous *in vivo *experiments have demonstrated higher endotoxin levels after bacterial challenge [16,17,23,24]. Furthermore, it has been shown that cachectin and TNF is associated with weight loss [25], which can be reversed by blocking TNF and IL-1 receptors [26]. TNF-α is a multifunctional cytokine that is secreted in response to injury, inflammation or infection. In thermal injury, TNF-α is considered a potent mediator of other cytokines such as IL-6 [25]. Clinical and experimental studies have shown a significant elevation in IL-6 production after burn injury and sepsis, which correlates with suppressed cell-mediated immunity and increased mortality [27-29]. In addition, blockade of IL-6 biological activity following burn injury and/or sepsis has been shown to improve outcome [30]. In our study we did not see any significant difference in IL-6 production. This is due, in part, to the limited burn area (30% BSA) without differences in mortality and development of burn-associated immunosuppression. A number of experimental studies have shown implication of Th-2 cytokines, like IL-10, in immunosuppression after thermal injury as well as in sepsis [31-33]. Recent findings have demonstrated that the macrophages are resistant to the suppressive effects of IL-10 post burn [34,35]. It has been verified that block of endogenous IL-10 enhances IL-6 and TNF-α release in response to LPS. Addition of exogenous IL-10 to the macrophages cultures suppresses inflammatory mediator release [36]. In this study we were able to show that IL-10 expression levels were associated with weight gain in animals with infected burn wounds.

## Conclusion

We hypothesize that infection control in burned patients is the most important factor for preventing burn-associated weight loss. Our results suggest that in the presence of infection, you get a SIRS response possibly due to transient endotoxemia that is only seen in the infection group. In contrast, both burn and infection get a late IL-10 (CARS) response, which is then associated with a return to normal weight in the infection group. More studies are clearly needed to fully understand the pathophysiology in infected burn wounds.

## Competing interests

The author(s) declare that they have no competing interests.

## Authors' contributions

LS, OB and MHF performed most of the experiments. LS, OB, MHF, FJ, ML, GS, AD, HUS, DR, SCW participated in the experimental design, data interpretation and writing of the manuscript

## Pre-publication history

The pre-publication history for this paper can be accessed here:


